# Pathology, Risk Factors, and Oxidative Damage Related to Type 2 Diabetes-Mediated Alzheimer's Disease and the Rescuing Effects of the Potent Antioxidant Anthocyanin

**DOI:** 10.1155/2021/4051207

**Published:** 2021-02-27

**Authors:** Muhammad Sohail Khan, Muhammad Ikram, Tae Ju Park, Myeong Ok Kim

**Affiliations:** ^1^Division of Life Science and Applied Life Science (BK 21), College of Natural Sciences, Gyeongsang National University, Jinju 52828, Republic of Korea; ^2^Haemato-oncology/Systems Medicine Group, Paul O'Gorman Leukaemia Research Centre, Institute of Cancer Sciences, MVLS, University of Glasgow, UK

## Abstract

The pathology and neurodegeneration in type 2 diabetes- (T2D-) mediated Alzheimer's disease (AD) have been reported in several studies. Despite the lack of information regarding the basic underlying mechanisms involved in the development of T2D-mediated AD, some common features of the two conditions have been reported, such as brain atrophy, reduced cerebral glucose metabolism, and insulin resistance. T2D phenotypes such as glucose dyshomeostasis, insulin resistance, impaired insulin signaling, and systemic inflammatory cytokines have been shown to be involved in the progression of AD pathology by increasing amyloid-beta accumulation, tau hyperphosphorylation, and overall neuroinflammation. Similarly, oxidative stress, mitochondrial dysfunction, and the generation of advanced glycation end products (AGEs) and their receptor (RAGE) as a result of chronic hyperglycemia may serve as critical links between diabetes and AD. The natural dietary polyflavonoid anthocyanin enhances insulin sensitivity, attenuates insulin resistance at the level of the target tissues, inhibits free fatty acid oxidation, and abrogates the release of peripheral inflammatory cytokines in obese (prediabetic) individuals, which are responsible for insulin resistance, systemic hyperglycemia, systemic inflammation, brain metabolism dyshomeostasis, amyloid-beta accumulation, and neuroinflammatory responses. In this review, we have shown that obesity may induce T2D-mediated AD and assessed the recent therapeutic advances, especially the use of anthocyanin, against T2D-mediated AD pathology. Taken together, the findings of current studies may help elucidate a new approach for the prevention and treatment of T2D-mediated AD by using the polyflavonoid anthocyanin.

## 1. Introduction

### 1.1. Obesity and Type 2 Diabetes

Over 40% of the world's population is overweight and obese, which is one of the crucial risk factors for insulin resistance, peripheral inflammation, and type 2 diabetes (T2D) [1]. The incidence of obesity is growing in the adult population in the United States, which is related to unhealthy lifestyle habits such as high calorie intake, inadequate exercise, and sedentary lifestyle [2]. Approximately 69.2% of American citizens are obese or overweight [3]. Alarmingly, the prevalence of obesity among young adults has been increasing rapidly.

Diabetic individuals may be more vulnerable to chronic type 2 diabetes mellitus (T2DM) and cardiovascular disorders (CVDs) [4]. Recent evidence suggests that obesity is an important risk factor for T2D. Obesity and diabetes share the same pathways of activation of inflammatory mediators, which play a role in insulin resistance and the pathogenesis of T2D. The pathogenesis of these disorders is associated with the number and phenotype of adipose tissue macrophages. A previous line of evidence suggested that adipose tissue macrophages release proinflammatory mediators to classically activate the M1 macrophages, which may contribute to insulin resistance and diabetes mellitus. In contrast, the adipose tissue from healthy individuals contains M2 phenotype inflammatory macrophages.

**Figure 1:**
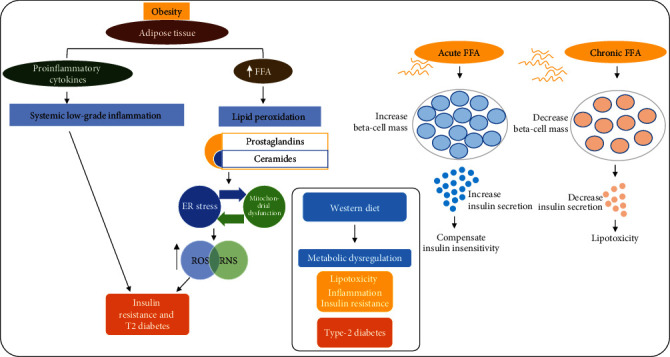
Obesity, free fatty acids, and insulin resistance. A diagrammatic representation showing the role of obesity in metabolic dysregulation via the release of inflammatory cytokines, oxidative stress, and lipotoxicity. Secretion of FFA and proinflammatory cytokines results in ROS/oxidative stress-mediated JNK activation. Inflammatory cytokines cause low-grade inflammation in peripheral organs and induce type 2 diabetes. Similarly, chronic FFA treatment induces insulin resistance and beta-cell dysfunction and causes type 2 diabetes. ROS: reactive oxygen species; RNS: reactive nitrosative stress; FFA: free fatty acid; ER: endoplasmic reticulum.

When fat cells in adipose tissue rupture, they usually produce a large quantity of free fatty acids (FFAs). Peripheral adipose tissues are also known to release various kinds of cytokines [5]. In addition to storing FFAs, excessive fat and adipose tissue also contain a large number of other compounds such as angiotensin-2, resistin, and inflammatory cytokines, such as TNF-*α*, IL-1*β*, and interleukin-6. The release of an excessive amount of FFAs in the circulation increases the plasma FFA level in obese individuals, and elevated plasma FFA levels prevent the antilipolytic action of insulin, which further increases FFA levels. The presence of a large quantity of FFAs in plasma also leads to peripheral insulin resistance [5–11]. Although FFAs are involved in increasing the mass of pancreatic beta-cells and improving insulin secretion to increase the availability of insulin to absorb the blood glucose derived from foods and via gluconeogenesis (metabolic process to obtain glucose from noncarbohydrate sources), a continuous and chronic increase in FFA levels can disrupt lipid metabolism, causing lipotoxicity, insulin resistance, decreased beta-cell function, and, eventually, T2D [12] (Figure 1).

### 1.2. Type 2 Diabetes Mellitus and Alzheimer's Disease

Several basic mechanisms are involved in the development of T2D. In one such mechanism, increased secretion of FFAs by adipose tissue in obese individuals induces the production of plasma malondialdehyde (MDA), which is a reactive product of lipid peroxidation. Increased lipid peroxidation subsequently induces the production of the toxic lipid ceramide and oxidative stress. The increased lipid peroxidation induced by FFAs also causes the release of prostaglandins. Moreover, a chronic increase in FFA levels induces insulin resistance and beta-cell dysfunction, mentioned previously. Thus, the reduction of elevated plasma FFA levels might be an important therapeutic target in obesity and T2D [13]. AGEs, which are also called mycotoxins, induce reactive oxygen species (ROS) and trigger the release of proinflammatory cytokines, causing sustained activation of innate immunity, via transcriptional factors such as nuclear factor-kappa B (NF-*κ*B) [14]. These prostaglandins and ceramides then activate the immune cells to secrete IL-1*β*, TNF-*α*, and IL-6.

Adipose tissues are also responsible for the release of proinflammatory cytokines and some other hormones. Increased release of proinflammatory cytokines can lead to the development of insulin resistance. Elevated oxidative stress causes lipid peroxidation and activation of several inflammatory signaling pathways [15], such as the c-Jun N-terminal kinase (JNK) and MAP kinase pathways, which leads to diabetes and pancreatic cell death [16–18]. Extensive research studies have shown that diabetes plays a significant role in the execution of Alzheimer's disease (AD) pathology [19]. Both conditions share many pathological features.

### 1.3. Anthocyanins and Their Effects against Obesity, Diabetes, and Alzheimer's Disease

**Figure 2:**
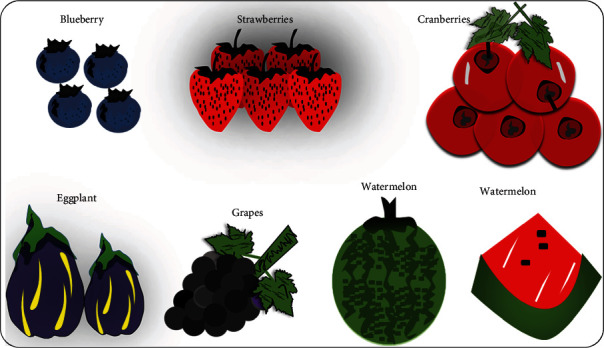
Rich sources of anthocyanins. Fruits and vegetables rich in the polyflavonoid anthocyanin.

Anthocyanins (a Greek word; from the flower) are a group of extensively studied natural compounds that belong to the subgroup of flavonoids [20]. They are water-soluble compounds that provide color to many fruits and vegetables; the blue color of various fruits and vegetables is attributable to the presence of anthocyanins in them. The ability of anthocyanins to form flavylium cations distinguishes them from other flavonoids. A total of 27 aglycones and 700 anthocyanins have been classified based on their chemical structures. Anthocyanins naturally occur in cherries, blueberries, strawberries, purple grapes, raspberries, black currants, and red wine [21]. The amount and composition of anthocyanins differ in different foods, varying from 0.1% to 1%. Foods that are rich sources of anthocyanins include blueberries, black soybeans, black plums, grapes, and black rice. Among the various types of fruits, berries are considered a rich source of anthocyanins (Figure 2).

Anthocyanins have recently gained tremendous interest because of their significant therapeutic roles in different diseases, most prominently metabolic and neurodegenerative diseases, since anthocyanin-rich foods have been used in traditional medicines [22]. Although these foods have been extensively used for the management of different diseases, little attention has been paid to their phytochemicals and the related mechanistic approaches. Studies have shown strong antioxidant and anti-inflammatory effects of anthocyanins against different neurodegenerative diseases. Several climatic factors (pH, light, and temperature), oxidation, and enzymolysis can affect the levels of anthocyanins. Recent findings have shown that anthocyanins are stable at low pH, i.e., in acidic conditions. Anthocyanins have been reported to show potent anti-inflammatory, antioxidant, anticancer, and antidiabetic activities [20, 22–33]. The daily consumption of natural dietary anthocyanin has been recently suggested to prevent various diseases and improve immune function [34–37].

## 2. Methodological Approaches

This review article is aimed at summarizing the findings of studies on the interaction of T2D and AD and the reported preventive and therapeutic effects of anthocyanins. The motivation for preparing this review was based on our previous studies on the role of anthocyanins against different neurodegenerative diseases (APP/PSEN-1 transgenic mice and LPS-treated mice). Here, we searched for potential research articles focusing on obesity, diabetes, and AD. In addition, to identify studies on the role of anthocyanins in obesity, diabetes, and AD, we conducted searches using the keywords “anthocyanins,” obesity,” and “diabetes” in all available and independent databases. For a clear understanding of these studies, the abstracts were fully studied and the main findings were recorded. For comparisons among the different groups, the anthocyanin-treated group was compared with the toxin-treated/model group. The dose and route of administration of anthocyanin, duration of treatment, and toxic compounds used in these studies were not considered. All studies covering animal and cellular models were included.

## 3. Risk Factors for Metabolic Dysfunction Induced by Type 2 Diabetes

### 3.1. Gut Dysbiosis and Type 2 Diabetes

As previously described, the presence of proinflammatory cytokines in various body tissues, particularly those that participate in metabolic regulation, such as muscles, livers, and adipose tissues, augments insulin resistance and T2D [38]. Growing evidence supports the concept that intestinal microbiomes play a critical role in the development of T2D, and patients with T2D have an altered gut microbiota. These findings have shown the gut microbiota to be one of the important contributors to the development of T2D. Changes in gut microbiota exaggerate systemic low-grade chronic inflammation, alter gut permeability, affect lipid and glucose metabolism, modulate insulin sensitivity, and disturb energy homeostasis. Several important mechanisms have been reported to describe the effects of gut microbiota on insulin resistance and T2D, of which metabolic endotoxemia is one of the most important. Recent studies have demonstrated that most gram-negative bacteria in the gut produce a very potent immunogenic endotoxin known as lipopolysaccharide (LPS). LPS is a component of the cell wall of gram-negative bacteria such as *E*. *coli* that causes metabolic endotoxemia. Obesity and increased fat intake induce the abundance of opportunistic gram-negative bacteria in the gut, which later produce LPS [39, 40].

**Figure 3:**
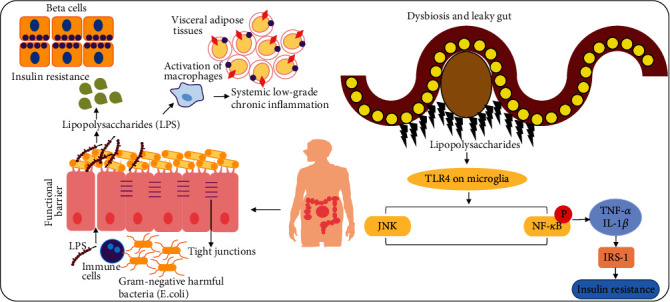
Dysbiosis, leaky gut, inflammatory cytokines, and insulin resistance. A simple illustration showing the release of lipopolysaccharides (LPS) and disruption of tight junctions in the intestinal wall resulting in the release of inflammatory cytokines, which enter the blood and pancreatic beta-cells. In addition, the TLR4 receptors in LPS play a role in the release of microglial cells. The reactive microglial cells secrete inflammatory cytokines and cause insulin resistance. NF-*κ*B: nuclear factor-kappa B; TNF-*α*: tissue necrosis factor-alpha; TLR4: Toll-like receptor 4.

Evidence from multiple studies indicates that opportunistic bacteria secrete LPS and other toxins inside the gut, abnormally increasing gut permeability by disrupting tight junction proteins [41]. This condition is also known as a leaky gut. A leaky gut allows several inflammatory cytokines and toxins to contaminate the blood and subsequently cause systemic inflammation. Furthermore, the presence of LPS in the gut causes epithelial cell inflammation. The secreted LPS further activates immune cells such as microglia and astrocytes. These cells secrete proinflammatory cytokines into the blood and promote their contamination. Once these proinflammatory cytokines enter the peripheral organs such as the liver, pancreas, and adipose tissue via the leaky gut, they cause peripheral inflammation, pancreatic cell dysfunction, insulin resistance, and T2D [41, 42]. Recent evidence suggests that Toll-like receptor 4 (TLR4) is a receptor for LPS present on the microglial surface. Once LPS binds to TLR4, it activates several signaling pathways such as the MAP kinase, NF-*κ*B, and inflammatory pathways [43, 44]. Activation of these pathways leads to the development of glucose intolerance and hepatic insulin resistance [45, 46] (Figure 3).

### 3.2. Lifestyle, Environmental Factors, and Diabetes

Physical inactivity, sedentary lifestyle, alcohol consumption, and smoking are common factors that play a role in the development of T2D. Treatable obstructive sleep apnea (OSA) and increased body weight are also considered important risk factors for T2D. Obstructive sleep apnea is more common in patients with T2D than in the general population. Similarly, diet and lack of exercise are other risk factors for T2D. Studies have shown that a diet with low fiber and high glycemic content is positively associated with T2D pathophysiology, while dietary fatty acid-rich foods may overcome the incidence of diabetes and insulin resistance [47–55]. Other studies have shown that saturated fatty acid-rich foods, sweets, and refined carbohydrates are potentially associated with an increased risk of diabetes. Furthermore, numerous observational studies have shown that the consumption of foods such as omega-6 industrial seed oils, cereal grains, and fructose in excessive quantities mediates peripheral inflammation and insulin resistance [56–60]. Fructose, also known as fruit sugar, is a simple ketonic monosaccharide found in many plants. There is increasing evidence that the excessive consumption of fructose, e.g., in the form of high-fructose corn syrup (HFCS) in soft drinks, may cause nonalcoholic fatty liver disease, which is strongly associated with diabetes and insulin resistance [61]. Apart from the fructose mentioned previously, some studies have suggested that saturated fatty acids (SFAs) are also hazardous in relation to these disorders. A palmitic acid-rich high-fat diet is associated with reduced sensitivity and enhanced atherosclerotic conditions, while a higher intake of eicosapentaenoic acid (EPA) and docosahexaenoic acid (DHA) may enhance insulin sensitivity. A detailed discussion may be found elsewhere [62]. Polyunsaturated fat and protein react with fructose and form AGEs. Recent studies provide compelling evidence for the idea that the complications that occur as a result of diabetes, such as neuropathy, nephropathy, cardiomyopathy, and retinopathy, are due to protein glycation and the formation of AGEs [63–67]. Diets with insufficient nutrients may affect the function of beta-cells. For example, a low-protein diet leads to the dysfunction of pancreatic cells. Moreover, several hormones such as stress hormones, such as adrenaline and cortisol, increase blood sugar by releasing glucose from the liver. Therefore, stress can directly increase blood glucose levels and affect body metabolism [68–73].

Viral infections can also directly damage the beta-cells or induce the release of proinflammatory cytokines. Upon exposure to the virus infection, the immune system of the pancreas becomes activated and initiates the release of inflammatory cytokines, which later cause beta-cell damage. The important viruses that are involved in the infection of pancreatic beta-cells are enterovirus, congenital rubella, and cytomegalovirus [74–77].

### 3.3. Drugs and Diabetes

Drugs such as cyclosporin, diphenylhydantoin, thiazide diuretics, and pentamidine are known to induce hyperglycemia-induced diabetes. Some experimental chemical agents such as streptozotocin and afloxan are also involved in the induction of diabetes. Numerous studies have indicated that the exogenous sources of advanced lipoperoxidation and glycation end products (ALEs and AGEs) are dairy products processed with sugar, roasted foods, and fried foods. Foods processed at high temperatures are a rich source of lipid-associated prooxidants and heat-accelerated proteins [78–84].

### 3.4. Shared Links between Diabetes and AD Pathology

An increasing body of evidence indicates that metabolic disorders increase the incidence of AD by disturbing glucose metabolism and its bioavailability in the brain [85]. The brain requires high amounts of glucose and oxygen for proper functioning. Therefore, the chances of the release of ROS will be greater in the brain than in other organs. Elevated oxidative stress by different mechanisms causes neuronal cell loss and neurodegeneration [86–88]. Oxidative stress plays a crucial role in the development of T2D-mediated Alzheimer's pathology. Recent studies have shown that chronic hyperglycemia produces oxidative stress in various organs, including the brain. Other studies have reported that hyperglycemia affects the antioxidant system and accelerates the production of free radicals in neuronal cells. Several other studies have demonstrated that persistent hyperglycemia in diabetic animals causes oxidative stress and disturbs the function of the antioxidant system. Diabetic encephalopathy, nowadays termed as “diabetes-associated cognitive decline” (DACD), is a serious brain disorder that is frequently observed in diabetic patients. Cognitive deficits and neuronal cell loss are common features of this disease. Hyperglycemia and oxidative stress are two important contributory factors in the development of diabetic encephalopathy. In the diabetic brain, hyperglycemia induces mitochondrial dysfunction and exaggerates oxidative damage. Oxidative stress produced as a result of excessive production of ROS in the diabetic brain mediates neuronal cell death and cognitive deficits [89–92].

**Figure 4:**
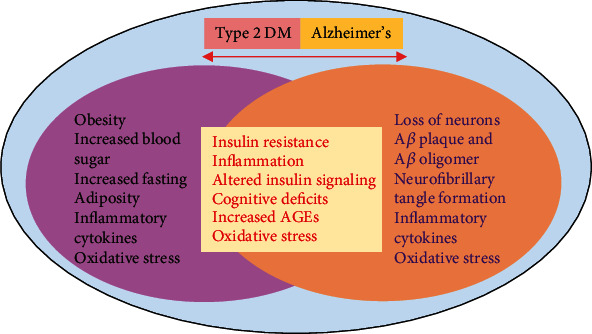
Shared links between Alzheimer's disease and diabetes. A simple illustration showing the shared links between Alzheimer's disease and diabetes.

Recent research has provided evidence that oxidative stress disturbs cellular proteins, nucleic acids, and lipids, which eventually interfered with the normal functions of the cell [93]. Oxidative stress activates an important stress kinase pathway known as the JNK pathway [94]. The JNK pathway is an important signaling pathway that plays a central role in the development of several neurodegenerative diseases such as AD and Parkinson's disease [95]. A large number of recent studies provide compelling evidence that JNK activates downstream NF-*κ*B signaling, which further activates the neuroinflammatory cascade and results in neuroinflammation [96]. Furthermore, NF-*κ*B has been shown to increase the activities of the BACE enzyme, which abnormally cleaves A*β* proteins and promotes the formation of amyloid-beta plaques [97, 98] (Figure 4).

Recent studies have shown that insulin resistance (IR) plays a crucial role in the progression of T2D pathology. No or very little response of a specific organ to the circulatory insulin is called IR. Loss of insulin binding receptor activities, dysregulation of the insulin receptor, and insulin receptor substrates (IRS-1 and IRS-2) are associated with an increased incidence of T2D pathology. Growing evidence indicates that peripheral IR disturbs brain insulin signaling [85]. Emerging studies have shown that increased circulatory insulin levels decrease the level of endothelial insulin receptors, impair the function of the blood-brain barrier (BBB), and decrease BBB insulin permeability [99].

**Figure 5:**
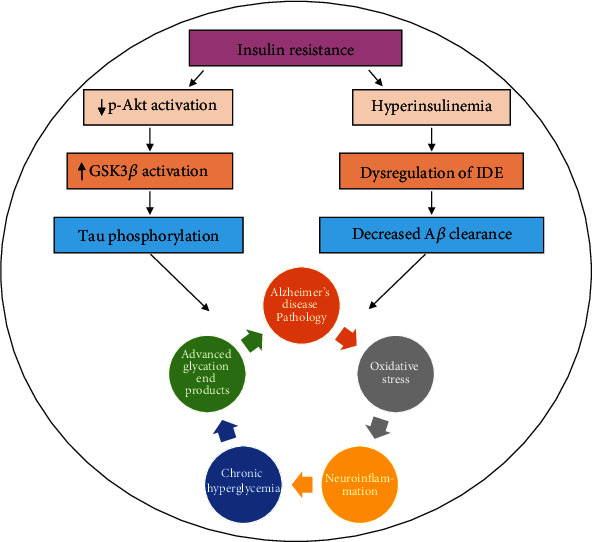
Insulin resistance, oxidative stress, and A*β* pathology. Downregulation of Akt and perturbation of the activities of the IDE lead to aggregation of amyloid-beta plaques and hyperphosphorylation of tau protein. On the other hand, chronic hyperglycemia directly promotes oxidative stress and neuroinflammation and causes neurodegeneration. A*β* = amyloid-beta; IDE = insulin-degrading enzyme; GSK3*β* = glycogen synthase kinase 3 beta.

A serine/threonine-specific protein kinase known as Akt is downregulated in IR. Akt facilitates glucose metabolism and inhibits glycogen synthase kinase 3 beta (GSK3*β*). GSK3*β* is an important kinase that phosphorylates tau protein. The overactivation of GSK3*β* in IR mediates the increased phosphorylation of tau protein, which later initiates the formation of neurofibrillary tangles. Moreover, another important enzyme called insulin-degrading enzyme (IDE) is dysregulated during IR. This enzyme usually prevents the accumulation of A*β* peptide in the brain via the degradation process [100, 101]. Several other pathways that have been observed in patients with AD and diabetes are inflammatory, oxidative stress, and protein misfolding signaling pathways. Numerous studies have reported that AGEs accumulate in the brain of a diabetic person. Recent evidence has demonstrated that dyslipidemia and hyperglycemia result in glucolipotoxicity, which plays a key role in the development of diabetes complications. Hyperglycemia also induces the production and release of AGEs, which act as an important link between diabetes and AD. The two known AGEs, pentosidine and glyceraldehyde-derived pyridinium (GLAP), have been identified to induce cognitive dysfunction and increase BACE1 activity. BACE1 is an enzyme that triggers the generation of A*β* by the activation of NF-*κ*B. The receptor for AGEs is RAGE, and these receptors also function for A*β*. Increased expression of RAGE has been observed in A*β*-treated cultured microglia. Other cells of the brain, such as neurons, astrocytes, and microvascular cells, also show an elevated level of RAGE during the A*β* burden and AD pathology. The levels of both RAGE and AGEs increased during the progression of AD pathology [19, 102–115]. A previous study showed that diabetes induced via streptozotocin elevates the level of RAGE, increases senile plaque formation, and enhances AD pathology in transgenic mice. Elevated levels of microglial RAGE have been found in AD transgenic mice, and this causes an increase in the production of proinflammatory cytokines. The elevated levels of proinflammatory cytokines in turn lead to an increase in A*β* plaque formation, tau hyperphosphorylation, and cognitive impairments [116–118] (Figure 5).

## 4. Beneficial Role of Anthocyanins against T2D-Mediated AD Pathology (Figure 6)

Adipose tissue hormones such as resistin, adiponectin, and leptin have regulatory effects on body metabolism. Several recent studies have reported that dysregulation or abnormal levels of these hormones (also known as adipokines) can cause metabolic disorders such as T2D. Increased levels of leptin and resistin in obese individuals have been shown to be responsible for the development of IR. Several studies have suggested that insulin may reduce T2D by different mechanisms. Hyperglycemia-induced ROS generation has been suggested to be a critical feature in the progression and pathogenesis of DM, since oxidative stress is an important factor responsible for the progression of DM [119]. An imbalance in the production of ROS and dysregulation in the antioxidant defense system are major factors in the pathophysiology of DM-associated disorders. Therefore, regulation of oxidative stress either by boosting the endogenous antioxidant mechanism [15] or by inhibiting oxidative stress may provide benefits against DM [120] and other neurodegenerative diseases [96, 121]. Previous studies have suggested that treatment of pancreatic *β*-cells with anthocyanins obtained from wild Chinese blueberries markedly reduced the ROS and upregulated the endogenous antioxidant defense mechanism, thereby regulating the hyperglycemia-induced glucolipotoxicity [122]. Another study conducted on streptozotocin-induced DM suggested that anthocyanins can potentially rescue DM-associated oxidative stress, since anthocyanins reduce the levels of oxidative stress-related markers such as malondialdehyde and restore superoxide dismutase and catalase activity in diabetic rats [123]. Studies conducted on rats showed that grape-bilberry juice rich in anthocyanins could reduce the levels of resistin and leptin. Additionally, grape-bilberry juice was shown to decrease the levels of SFAs and increase the proportion of polyunsaturated fatty acids in the rat plasma. In conclusion, the authors suggested that anthocyanins could largely prevent metabolic diseases such as T2D and IR [124, 125].

Increasing the level of beneficial bacteria and regulating gut dysbiosis is another approach that may tackle DM-associated complications, since beneficial bacteria in the gut not only participate in the synthesis of several vitamins and amino acids but also ferment complex carbohydrates to produce short-chain fatty acids (SCFAs). Several studies have indicated that anthocyanins could increase the levels of beneficial bacterial species such as *Bifidobacterium* and *Lactobacillus*-*Enterococcus* [126–128]. A wealth of emerging studies in recent years indicated that abnormal glucose metabolism produces oxidative stress in body cells, which plays a pivotal role in the development of T2D. Several vitamins such as vitamin B complex, vitamin C, and vitamin K have antioxidant properties that abrogate oxidative stress and regulate glucose metabolism. Furthermore, these vitamins also reduce the risk of T2D in prediabetic individuals [129–133].

Accumulating recent evidence has highlighted that hyperglycemia and IR are two important features of T2D and that hyperglycemia mediates neuroinflammation and induces oxidative stress. Increased oxidative stress activates several neuronal apoptotic pathways and causes neurodegeneration. Furthermore, increased oxidative stress and neuroinflammation are involved in the pathogenesis of AD. Anthocyanins not only stimulate glucose metabolism but also reduce hyperglycemia and thus prevent diabetes-induced neuroinflammation and AD pathology [134–144].

Insulin resistance causes hyperinsulinemia in the body, which in turn leads to dysregulation of IDEs, which are important enzymes involved in the clearance of A*β* proteins. Dysregulation of IDEs causes an A*β* burden in the brain, and excessive accumulation of A*β* proteins induces neurodegeneration and accelerates AD pathology. Emerging evidence suggests that the daily consumption of the natural dietary polyflavonoid anthocyanin increases insulin sensitivity, regulates insulin signaling, and prevents insulin resistance-mediated AD pathology. Moreover, anthocyanin enhances the function of insulin-secreting pancreatic *β*-cells and reduces the risk of diabetes-induced AD pathology [122, 145–150]. The LPS secreted by gram-negative bacteria during gut dysbiosis has been shown to cause systemic low-grade inflammation, pancreatic cell dysfunction, IR, and high levels of cytokines in the blood. Circulatory cytokines and LPS affect the function of peripheral organs and enter the brain via the BBB, causing neuroinflammation, neuronal cell death, and A*β* pathology. A recent report has shown that anthocyanins have strong antioxidant and anti-inflammatory properties. Daily intake of anthocyanins improves the growth of beneficial bacteria in the gut and prevents the induction of peripheral as well as central nervous system (CNS) inflammation. Additionally, anthocyanins can also reduce the prevalence of T2D and its related complications. Several recent studies have suggested that anthocyanins could prevent diabetic neuropathy, brain IR, and AD pathology [151–164].

## 5. Conclusions and Future Perspectives

Type 2 diabetes is one of the major health-related challenges worldwide. Excessive consumption of fast foods and sedentary lifestyles are the most common causes of T2D. Lack of physical activity influences the hormone levels and induces obesity, which may further aggravate the progression of DM and its associated pathological consequences via several mechanisms. On the other hand, contagious diseases and contaminated foods increase the usage of broad-spectrum antibiotics, and prolonged usage of these drugs induces gut dysbiosis and reduces the abundance of beneficial bacteria, which are largely involved in the synthesis of multivitamins in the body. These multivitamins protect the body from oxidative stress-mediated metabolic diseases such as diabetes, abnormal weight gain, impaired insulin signaling, and AD-like pathological consequences. Various phytonutrients have drawn much attention in the management of DM-associated disorders such as neurodegeneration and neuroinflammation [96, 165]. One such phytonutrient is anthocyanin, a polyflavonoid that shows protective effects via different mechanisms, especially by reducing oxidative stress and boosting the endogenous antioxidant system [22]. Patients with diabetes are at a high risk of developing AD and dementia. This comprehensive study suggests that the daily consumption of natural dietary anthocyanins obtained from fruits, vegetables, and beans protects our bodies from various metabolic and neurological disorders. We believe that continuous research and detailed analyses will further elucidate the “dark side,” i.e., the basic molecular mechanisms underlying the effects of anthocyanins in diabetes and metabolic disease-mediated AD pathology, and diabetes patients will soon “wind down with red wine, pop in the purple potatoes, grab the grapes, and revel with radishes.”

## Figures and Tables

**Figure 6:**
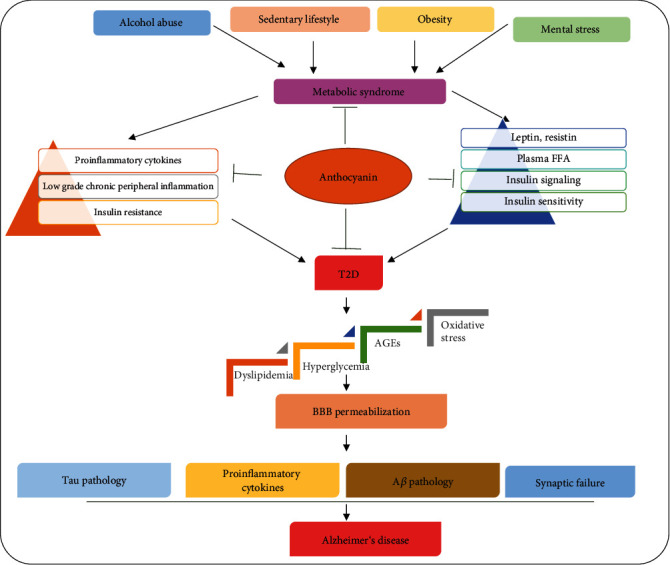
The effects of sedentary lifestyle, obesity, alcohol consumption, and mental distress on insulin sensitivity, induction of inflammation, release of free fatty acids, oxidative stress, hyperglycemia, neuronal insulin resistance, and AD pathology. T2DM = type 2 diabetes mellitus; A*β* = amyloid-beta; FFA = free fatty acid; BBB = blood-brain barrier.

## Data Availability

The datasets used and/or analyzed during the current study are available from the corresponding author upon request.

## Abbreviations

GLAP: Glyceraldehyde-derived pyridinium
AD: Alzheimer's disease
CNS: Central nervous system
IL-1*β*: Interleukin-1*β*
LPS: Lipopolysaccharide
MDA: Malondialdehyde
p-JNK: Phospho-c-Jun N-terminal kinase 1
T2D: Type 2 diabetes
AGEs: Advanced glycation end products
FFA: Free fatty acid
TNF-*α*: Tumor necrosis factor-*α*
ROS: Reactive oxygen species
*E. coli*: *Escherichia coli*
TLR4: Toll-like receptor 4
NF-*κ*B: Nuclear factor kappa-light-chain-enhancer of activated B cells
GIT: Gastrointestinal tract
OSA: Obstructive sleep apnea
DACD: Diabetes-associated cognitive decline
IR: Insulin resistance
IRS: Insulin receptor substrates
BBB: Blood-brain barrier
SCFAs: Short-chain fatty acids.
